# Supplementary material for: Physician’s use of sickness certification guidelines: a nationwide survey of 13 750 physicians in different types of clinics in Sweden
Source: BMJ Open. 2021 Dec 13;11(12):e051555. doi: 10.1136/bmjopen-2021-051555 (PMC8672016; doi:10.1136/bmjopen-2021-051555)
Supplement: Author's manuscript [file bmjopen-2021-051555.draft_revisions.pdf]

BMJ Open is committed to open peer review. As part of this commitment we make the peer review history of every article we publish publicly available.

When an article is published we post the peer reviewers' comments and the authors' responses online. We also post the versions of the paper that were used during peer review. These are the versions that the peer review comments apply to.

The versions of the paper that follow are the versions that were submitted during the peer review process. They are not the versions of record or the final published versions. They should not be cited or distributed as the published version of this manuscript.

BMJ Open is an open access journal and the full, final, typeset and author-corrected version of record of the manuscript is available on our site with no access controls, subscription charges or pay-per-view fees (http://bmjopen.bmj.com).

If you have any questions on BMJ Open's open peer review process please email info.bmjopen@bmj.com

BMJ Open

Physician's use of sickness certification guidelines: a nationwide survey of 13 750 physicians in different types of clinics in Sweden

Journal:	*BMJ Open*
Manuscript ID	bmjopen-2021-051555
Article Type:	Original research
Date Submitted by the Author:	22-Mar-2021
Complete List of Authors:	Svärd, Veronica; Karolinska Institutet, Division of Insurance Medicine, Department of Clinical Neuroscience; Södertörn University, Department of Social Work<br>Alexanderson, Kristina; karolinska institutet, Department of Clinical Neuroscience
Keywords:	Health policy < HEALTH SERVICES ADMINISTRATION & MANAGEMENT, PUBLIC HEALTH, SOCIAL MEDICINE, Protocols & guidelines < HEALTH SERVICES ADMINISTRATION & MANAGEMENT

SCHOLARONE™
Manuscripts

I, the Submitting Author has the right to grant and does grant on behalf of all authors of the Work (as defined in the below author licence), an exclusive licence and/or a non-exclusive licence for contributions from authors who are: i) UK Crown employees; ii) where BMJ has agreed a CC-BY licence shall apply, and/or iii) in accordance with the terms applicable for US Federal Government officers or employees acting as part of their official duties; on a worldwide, perpetual, irrevocable, royalty-free basis to BMJ Publishing Group Ltd ("BMJ") its licensees and where the relevant Journal is co-owned by BMJ to the co-owners of the Journal, to publish the Work in this journal and any other BMJ products and to exploit all rights, as set out in our licence.

The Submitting Author accepts and understands that any supply made under these terms is made by BMJ to the Submitting Author unless you are acting as an employee on behalf of your employer or a postgraduate student of an affiliated institution which is paying any applicable article publishing charge ("APC") for Open Access articles. Where the Submitting Author wishes to make the Work available on an Open Access basis (and intends to pay the relevant APC), the terms of reuse of such Open Access shall be governed by a Creative Commons licence – details of these licences and which Creative Commons licence will apply to this Work are set out in our licence referred to above.

Other than as permitted in any relevant BMJ Author's Self Archiving Policies, I confirm this Work has not been accepted for publication elsewhere, is not being considered for publication elsewhere and does not duplicate material already published. I confirm all authors consent to publication of this Work and authorise the granting of this licence.

For peer review only

Physician's use of sickness certification guidelines: a nationwide survey of 13 750 physicians in different types of clinics in Sweden

Veronica Svärd[1, 2] & Kristina Alexanderson[1]

[1] The Division of Insurance Medicine, Department of Clinical Neuroscience, Karolinska Institutet, SE-171 77 Stockholm, Sweden

[2] Department of Social Work, Södertörn University, SE-141 89, Huddinge, Sweden

Correspondance to Dr Veronica Svärd; veronica.svard@ki.se

Word count: 3441

For peer review only - http://bmjopen.bmj.com/site/about/guidelines.xhtml

ABSTRACT

Objectives: To explore physicians' experiences of using the national sickness certification guidelines introduced in 2007, the types of information they used, in general and in different types of clinics.

Design: Cross-sectional survey.

Setting: Most physicians working in Sweden in 2017.

Participants: A questionnaire was sent to 34 718 physicians; 54% responded. Analyses were based on answers from the 13 750 physicians who had sick-leave cases.

Outcome measures: To what extent the guidelines were used and what type of information from them that was used.

Results: Ten years after the sickness certification guidelines were introduced in Sweden, half of the physicians used them at least once a month. About 40% of physicians in primary healthcare and occupational health services used the guidelines every week. The type of information used varied; 53% used recommendations about duration and 29% about degree of sick leave. Using information about function and activity/work capacity, respectively, was more common within primary healthcare (37% and 38%), psychiatry (42% and 42%), and occupational health services (35% and 41%), and less common in surgery and orthopedic clinics (12% and 12%) who more often used information about duration (48% and 53%). Moreover, 10% stated that the guidelines were very, and 24% fairly problematic to apply. Half (47%) stated that the guidelines facilitated their contacts with patients and 29% that they improved quality in their management of sick-leave cases. Non-specialists found that the guidelines facilitated contacts with patients (odds ratio: 3.28 (95% confidence interval: 3.04-3.55)), to a higher extent than specialists.

For peer review only

Conclusions: The majority of the physicians used the sickness certification guidelines, although this varied with type of clinic. Half stated that the guidelines facilitated patient contacts. Yet, some found it problematic to apply the guidelines. Further development of the guidelines is warranted as well as more knowledge about them among physicians.

Keywords: sick leave, sickness certification, physician, guidelines, clinics

For peer review only

STRENGTHS AND LIMITATIONS OF THIS STUDY

- With 13 750 responding sick-listing physicians this is the largest survey of physicians' work with sick-leave cases, also internationally, allowing several types of subgroup analyses.
- All physicians involved in sickness certification were included, not only GPs, which is seldom the case in this research area.
- Although the response rate (54%) was relatively high for this type of study, we do not know if non-responders would have responded differently.
- Comparisons with findings from the few studies from other countries is problematic due to large differences in both sickness absence insurance systems and in available sickness certification guidelines.
- The cross-sectional design means that no conclusions about causality can be drawn.

For peer review only - http://bmjopen.bmj.com/site/about/guidelines.xhtml

INTRODUCTION

In Sweden, the sick-leave rates have fluctuated much in the last decades,[1] and many interventions have been initiated to keep the rates at a more stable and lower level. Several of the interventions have been directed towards physicians, who have a central role in the sick-leave process. Several studies and systematic reviews have found that physician's find aspects of sickness certification (SC) of patients as problematic,[2-6] e.g., the following aspects: their own relative lack of competence regarding insurance medicine, lack of support for handling sickness certification, and the general lack of scientific knowledge regarding optimal sick leave and handling of such work tasks.[2-6] This is one of the reasons for why different types of SC guidelines have been asked for and, in some countries also introduced.[7-11] In Sweden, such guidelines were introduced in 2007.

SC guidelines are to assist clinicians in their handling of consultations regarding sickness absence. These types of guidelines serve, when they exist, as recommendations for clinicians to uphold the best practice regarding SC. Some of the guidelines that have been introduced are, however, merely based on data regarding duration of previous sick-leave cases,[10] which involves several problems when put in practical use. An essential barrier to develop guidelines in Sweden as well as in other countries, is the lack of scientific knowledge regarding the need for, as well as possible consequences/side effects of being on sick leave for different periods of time among patients with specific medical conditions. Moreover, such knowledge would be needed in relation to different types of work task the patient has, as it is not a diagnosis in itself that gives the right to be on sick leave but the extent to which the diagnosis limits functions that are of importance for that individual's work capacity – that is, in relation to his/her specific work tasks.

Due to the limited number of studies regarding optimal and/or needed sick leave among people with different medical conditions and work tasks, such guidelines so far cannot be

For peer review only - http://bmjopen.bmj.com/site/about/guidelines.xhtml

based on scientific evidence.[8] Although Letrilliart and Barraur in a systematic review about issues regarding SC, argued that one strategy to improve sick-listing practice would be to provide guidelines, they found that general practitioners (GP) in Europe demonstrate low awareness and use of guidelines, and that the guidelines are ineffective without training in how to use them.[9]

Most studies regarding physicians' SC practice have so far focused on GPs.[2 4-6] However, in Sweden, as in many countries, all physicians, not only GPs can issue sick-leave certificates. In Sweden, such a certificate is needed after the 7th day of a sick-leave spell. The sickness certificate is used by the officers of the Social Insurance Agency to assess whether the patient/claimant fulfills the criteria for sick-leave benefits, which can be granted for full- or part-time (100, 75, 50, or 25%) of ordinary work hours. However, as clear from above, there is a nearly total lack of scientific studies on optimal sick-leave duration and degree for patients with specific diagnoses and work tasks. Therefore, the SC guidelines for specific diagnoses were developed in Sweden by the National Board of Health and Welfare, based on discussions in expert groups, rather than based on established scientific evidence. For many diagnoses and for less severe conditions, the recommendation in the guidelines was no sick leave at all, or for a few days or weeks. For other diagnoses, longer sick-leave spells could be accepted, especially if the patient had a physically demanding job. After a pilot-study, that fell well out, the Swedish National Board of Health and Welfare issued the web-based nationwide SC guidelines in 2007. They consist of two sets of recommendations: general guidelines covering the principles related to sickness certification and the diagnosis-specific recommendations.[12] The latter includes recommendations for duration and degree (full- or part-time) of sick leave as well as diagnosis-specific information about assessments of function, activity, and work capacity.[13] They cover about 110 of the most prevalent sick-leave diagnoses and are continuously updated.

For peer review only - http://bmjopen.bmj.com/site/about/guidelines.xhtml

Previous studies about physician's use of these SC guidelines in Sweden show that a large majority (>85%) of GPs who have used guidelines find them beneficial to ensure high quality in handling sick-leave cases.[14] The use of guidelines among GPs[14] and among gynecologists/obstetricians[15] have increased over the years, as did the rate of gynecologists/obstetricians who reported that the guidelines facilitated their contacts with patients.[15] Another study compared the quality of sickness certificates before and after the SC guidelines were introduced, in 2007 respectively 2009. Improvement of the quality of the information provided in the certificates was found regarding descriptions on functioning - although body impairments still dominated the descriptions of functioning, contradictory to the SC guidelines emphasizing of that functioning should be described in terms of activity and work incapacity.[16] These previous studies do not, however, provide any knowledge about what type of information from the guidelines physicians find useful, and if this differs between type of clinic, such as psychiatry, orthopedics, and primary healthcare. Therefore, the aim of this study was to explore physicians' experiences of using the nationwide SC guidelines, what kind of information in the guidelines they used, and how this differed with type of clinic.

METHODS

Analyses of answers to a questionnaire sent to 34 718 physicians working and living in Sweden.

The questionnaire

A questionnaire about physician's work with sick-leave consultation including 133 questions was developed, based on previous surveys (2004, 2008, and 2012).[17-20] The items in the previous questionnaires were based on results from interviews, pilot studies, and other

For peer review only - http://bmjopen.bmj.com/site/about/guidelines.xhtml

studies.[2 21] Minor revisions of the 2012 questionnaire were made, based on previous results and discussions with physicians as well as other stakeholders.

The questionnaire included questions about frequency of sick-leave consultations, frequencies and severity of experienced problems, organizational prerequisites, internal and external cooperation, competence in insurance medicine, and about the use of the Swedish SC guidelines – the latter was the focus of this study.

Participants

The questionnaire was sent to all the physicians, 68 years and younger, living and working in Sweden in 2017, identified from the Swedish Healthcare Address Register of all physicians working in Sweden, administrated by QuintilesIMS. Specialists working in clinics that seldom have sick-leave cases were excluded, e.g., in laboratory, radiology, forensic, geriatric, and pediatric clinics. Statistics Sweden administrated the data collection, to ensure the respondents anonymous participation, and delivered an anonymized data file to the project group, including information on sex and age.

The questionnaire and a prepaid response envelope were sent to the participant's home addresses together with a cover letter with information about the project and that participation was voluntary. The questionnaire could be answered either over the web or by paper. Four reminders were sent to those who had not yet responded.

The study population included 34 718 physicians and 18 714 of them responded (54.1% response rate). As usual in surveys, the response rate was somewhat higher among women and older physicians.

Statistical analysis

Descriptive statistics were used. Variables about the use of the SC guidelines were analyzed and comparisons were made between types of clinics regarding the following items:

For peer review only - http://bmjopen.bmj.com/site/about/guidelines.xhtml

For peer review only

educational level, type of clinic, frequency of sick-leave cases, use of the guidelines, and if the guidelines were experienced as useful. Further variables concerned what information in the guidelines that were used: recommendations about duration and degree, information about function, activity/work capacity, or none. Logistic regression was used to calculate odds ratios (OR) with 95% confidence intervals (CI) for analyzes of how the guidelines facilitated physicians' contacts with patients.

In total, 13 750 physicians (78% of the respondents) had sick-leave cases at least once a year and those were included in the study. Those not specifying level of education (0.2% of all) or main type of clinic (0.3%, n=46) were not included analyses related to level of education and type of clinic, respectively. For the specific questions about the SC guidelines, answers were missing for 2.2 – 4.9% of respondents and for one question 7.3%. Those were not included in the analyses of the respective question.

RESULTS

Of the sick-listing physicians, half (50.4%; n=6929) were women and 68% were board-certified specialists. A third (34%) worked in primary healthcare (Table 1), where the proportion of board-certified specialists was somewhat lower (61%). The highest proportions of physicians having sick-leave cases at least six times a week were in falling order in orthopedics (74%), occupational health services (72%), rehabilitation (69%), oncology (61%), psychiatry (58%), pain (54%), and primary healthcare (45%).

Half (50%) used the SC guidelines at least once a month and 26% at least once a week. The frequency in use varied with type of clinic; physicians in occupational health services used the guidelines most often; 18% at least six times a week and 42% at least once a week. This was expected as all their patients are of working age and employed. About the same proportion of the GPs (41%) used the guidelines at least once a week.

For peer review only - http://bmjopen.bmj.com/site/about/guidelines.xhtml

Those who had sick-leave cases often also used the guidelines more often (Figure 1). The proportion (22-23%) who did not use the guidelines were the same irrespective if the frequency of sick-leave cases were 1-5 times a week or more than 6 times a week. Among those who had such consultations less than once/week, 40% stated that they did not use the guidelines.

For peer review only

For peer review only - http://bmjopen.bmj.com/site/about/guidelines.xhtml

Table 1. Number and proportion (%) of sick-listing physicians, proportions (%) of frequency of sick-leave cases, of frequency in use, and of types of information in the sickness certification guidelines used, by type of clinic (falling order according to size).

			Frequency of sick-leave cases			Frequency in using the guidelines					Type of information used from the guidelines					
Type of clinic	n	%[1]	≥6 cases a week	1-5 cases a week	Monthly	At least 6 times a week	1-5 times a week	Monthly	A few times a year	Never /almost never	None	Duration	Part- /full-time	Function	Activity /work capacity	Other
Primary healthcare	4687	34.1	45	50	4	9	32	31	18	10	18	69	43	37	38	3
Internal medicine	1661	12.1	12	46	41	2	10	22	34	32	37	53	28	19	18	2
Surgery	1285	9.3	27	52	21	4	13	18	27	38	46	48	18	12	12	1
Psychiatry	1050	7.6	58	32	10	11	23	27	20	19	28	45	29	42	42	3
Other	1029	7.5	33	20	36	4	11	18	24	43	44	37	20	16	17	1
Orthopedic	921	6.7	74	23	3	11	21	20	21	27	39	53	15	12	12	2
Ear, nose and throat	421	3.1	9	56	35	0	3	13	30	53	52	38	17	14	14	2
Infection	357	2.6	9	55	36	1	7	18	34	41	44	50	20	10	10	0
Oncology	342	2.5	61	31	8	5	11	20	25	39	50	34	13	16	15	2
Eye	341	2.5	3	19	78	1	3	8	28	60	67	25	9	8	9	1
Occupational health services	338	2.5	72	20	8	18	24	22	22	13	22	59	36	35	41	4
Gynecology	320	2.3	21	51	28	5	19	22	26	27	37	45	33	18	21	1
Neurology	305	2.2	29	58	13	1	12	16	35	36	46	38	23	20	19	2
Rheumatology	198	1.4	24	61	16	2	13	17	31	37	46	36	23	23	24	3
Skin	171	1.2	4	8	88	1	2	6	37	54	59	35	15	11	12	1
Rehabilitation	158	1.1	69	23	7	10	14	15	35	25	40	40	18	23	25	3
Administration[2]	66	0.5	9	30	60	3	10	13	25	49	45	39	24	18	26	2
Pain	54	<0.5	54	33	13	10	4	14	24	47	54	22	17	24	19	4
**All physicians**	**13 750**	**100**	**38**	**43**	**19**	**6**	**20**	**24**	**24**	**26**	**33**	**53**	**29**	**25**	**26**	**2**

[1]Column percentage, all other row percentages.

[2]Including research and education (i.e., physicians mainly working with education, research, or as managers but also having patients some of the time).

Regarding what type of information from the guidelines the physicians used, more than half (53%) used recommendations about duration of sick leave, while 29% used recommendations about degree (part- or full-time sick leave) (Table 1). Half or more of the physicians within primary healthcare (69%), occupational health services (59%), internal medicine (53%), orthopedics (53%), and infection (50%) used the recommendations about duration. Using recommendations about degree was most common in primary healthcare (43%), gynecology (33%), and occupational health services (36%). Use of the information about limitation of function or about activity limitation/work capacity was more common within primary healthcare (37% and 38%, respectively), psychiatry (42% and 42%), and occupational health services (35% and 41%). A majority of the physicians within eye (67%), skin (59%), oncology (50%), pain (54%), and ear, nose, and throat clinics (52%) did not use any information from the guidelines, partly due to lack of SC guidelines regarding several diagnoses handled in those clinics. Also, the proportions varied *within* types of clinics regarding type of information used. While about half of physicians within surgery and orthopedics used information about duration, only 12% used information about limitation of function or activity/work capacity (Table 1). Within psychiatry, on the other hand, almost the same proportions used information about limitation of function or activity/work capacity (42%) as recommendations about the duration (45%).

Ten percent of those who used the guidelines stated that the they were very problematic to apply and 24% that they were fairly problematic to apply, while 25% never found them problematic to apply. A somewhat higher proportion of those who used the guidelines less than once a week (11%) compared to at least once a week (8%) stated that they were very problematic to use (Figure 2).

Nearly a third (29%) reported that the guidelines to a high extent improved the quality in their management of SC; and higher proportions in gynecology (35%) and internal medicine (33%)

For peer review only - http://bmjopen.bmj.com/site/about/guidelines.xhtml

stated this, compared to, e.g., in the clinics with fewer physicians such as rehabilitation (17%) and pain clinics (19%) (Table 2).

Table 2. Proportion (%) of physicians who stated that the sickness certification guidelines are important for good quality in her/his handling of sickness certification and facilitate their contacts with patients or others, by type of clinic.

	The extent to which guidelines is of importance for the quality of how I handle sickness certification			The guidelines facilitate my contacts with ...					
Type of clinic	Very	Moderately	None	The patient	Social Insurance Agency	Health care staff	Employer	Employ-ment office	At least one of all
Primary healthcare	31	57	13	55	45	33	23	25	65
Internal medicine	33	51	16	41	41	31	22	22	53
Surgery	30	49	21	46	34	28	18	17	48
Psychiatry	30	54	16	40	43	31	22	25	55
Other	30	45	25	35	35	30	19	20	39
Orthopedic	24	51	25	44	35	24	16	16	50
Ear, nose and throat	23	56	22	39	31	22	17	18	41
Infection	32	54	15	44	35	23	18	16	46
Oncology	24	55	21	35	37	20	13	13	42
Eye	21	56	23	26	25	25	13	14	27
Occupational health services	22	62	16	46	37	24	31	17	58
Gynecology	35	51	15	48	37	29	19	16	51
Neurology	21	59	20	31	32	17	12	14	42
Rheumatology	21	49	30	34	33	24	19	19	41
Skin	26	54	20	40	27	24	15	16	40
Rehabilitation	17	63	20	32	30	22	19	19	42
Administration	21	55	23	33	36	18	18	16	45
Pain	19	57	31	33	28	28	18	20	33
**All physicians**	**29**	**54**	**17**	**47**	**39**	**29**	**21**	**21**	**53**

Half (53%) stated that the guidelines facilitated their contacts with at least one of the suggest; i.e., patient, Social Insurance Agency, healthcare staff, workplace and/or employment office. About twice as high proportions in primary healthcare (65%) and occupational health services (58%) stated this compared to among the physicians in smaller clinics that seldom used the guidelines, such as eye clinics (27%). Lower proportions stated that the guidelines facilitated their contacts with workplaces or employment offices (21%) and healthcare staff (29%)

For peer review only - http://bmjopen.bmj.com/site/about/guidelines.xhtml

compared to with the Social Insurance Agency (39%) and patients (47%). However, this varied with type of clinic. Here it is important to remember that all physicians had contacts with their patients, while only a few had contacts with the other stakeholders, which is why the figures for those are expected to be lower. A third (31%) of physicians in occupational health services stated that the guidelines facilitated their contacts with workplaces. Among those mainly working in oncology (37%), psychiatry (43%), or neurology (32%), the highest proportions stated that the guidelines facilitated their contacts with the Social Insurance Agency. For most type of clinics, however, the highest proportions reported that the guidelines facilitated their contacts with the patient.

The ORs for stating that the SC guidelines facilitated the contact with the patient was higher among women (OR 1.23 (95% CI 1.15-1.32) then men, among younger physicians (OR aged 24-39: 3.13 (2-86-3.42); OR aged 40-54: 1.38 (1.26-1.51)), compared to the older physicians (aged 55-68) (Table 3). Also, the OR regarding this was more than three times as high for non-specialists (OR 3.28 (3.04-3.55)), compared with specialists. As shown in Table 2, a higher rate of the physicians in primary healthcare stated that the guidelines facilitated contacts with patients (55%). In Table 3, the ORs for stating this is shown for four other specific clinical settings and for the rest of the physicians, using GPs as reference group. All the ORs are statistically significant, also when adjusting for proportions of specialist in the different groups. Thus, it seems as the GPs to a larger extent find the SC guidelines useful in this respect. When restricting those analyses to physicians who at least once a month used the guidelines, the ORs were about the same, with one exception: physicians in internal medicine (adjusted OR 1.42 (1.10-1.83).

Table 3. Crude and adjusted odds ratio (OR) with 95% confidence intervals (CI) for stating that the sickness certification guidelines facilitate the communication with patients, in different type of clinics. Among all physicians and among physicians who at least once a month used the guidelines.

For peer review only - http://bmjopen.bmj.com/site/about/guidelines.xhtml

Type of clinic	Crude OR	95% CI	Adjusted OR	95% CI
All				
Primary healthcare	Ref		Ref	
Internal medicine	0.84	0.75-0.94	0.86	0.76-0.97
Psychiatry	0.56	0.49-0.65	0.61	0.53-0.70
Orthopedic	0.65	0.56-0.75	0.73	0.63-0.85
Occupational health services	0.72	0.58-0.90	1.07	0.85-1.34
All other	0.55	0.51-0.60	0.59	0.54-0.64
Used the guidelines at least monthly				
Primary healthcare	Ref		Ref	
Internal medicine	1.54	1.20-1.97	1.42	1.10-1.83
Psychiatry	0.58	0.44-0.75	0.58	0.45-0.76
Orthopedic	0.93	0.67-1.28	0.95	0.68-1.31
Occupational health services	0.70	0.44-1.13	0.86	0.54-1.39
All other	1.21	1.01-1.44	1.15	0.95-1.38

[1] Adjusted for number of specialists.

DISCUSSION

In this study, a very large number (13 750) of the sick-listing physicians in Sweden responded to questions about their use of the SC guidelines, ten years after the guidelines were introduced in Sweden in 2007. Half (50%) used the guidelines at least once a month and 26% at least once a week, although those rates differed much with type of clinic and also regarding what type of information the physician used. Half (47%) stated that the guidelines facilitated their contacts with patients and 29% that they improved quality in their management of sickness certification cases. Ten percent stated that the guidelines were very problematic to apply and 17% did not experienced that the guidelines improved the quality of their handling of SC.

Comparing results with those from previous Swedish studies shows that the every week use increased among GPs from 22% in 2008[11] and 29% in 2012[14] to 41% in 2017. Among occupational health physicians, every week use increased from 33% in 2008[22] to 42% in 2017. Internationally, there are hardly no equivalent studies, however, in UK it was found that four years after introduction of SC guidelines, 36% of the 77 responding GPs were aware of

For peer review only - http://bmjopen.bmj.com/site/about/guidelines.xhtml

the guidelines and 20% of these 77 GPs used them.[23] This might be compared to the 76% of the responding GPs who used guidelines at least a few times a year already one year after the guidelines were introduced in Sweden in 2007.[11] While Letrilliart and Barraurs systematic review found that GPs in Europe demonstrate low awareness and use of SC guidelines,[9] our findings show that this is not the case in Sweden. However, precise comparisons cannot be made between the studies conducted in different European countries, because of different selections, contexts, and regulations, yet they show that differences between the countries exists. One reason for the high awareness and use of the SC guidelines in Sweden might be that the Social Insurance Agency use the guidelines when assessing claimant's right to sick-leave benefits. The relatively high use among physicians in occupational health services should be related to that they only have employed patients, while other physician to a larger extent have patients who not are in paid work, or not even of working age.

For some groups of diagnoses there are fewer diagnosis-specific SC guidelines than for other groups, which probably explains some of the large variations between different type of clinics' use of the guidelines. Another finding was that the type of information used varied somewhat with type of clinic. In psychiatry, a comparatively high proportion used information about function and activity/work capacity, perhaps meaning these guidelines were particularly well written, or that psychiatrists need such information more than other physicians. In other type of clinics, such as internal medicine, neurology, gynecology, and pain, information about function limitations were used to a higher extent than information about activity/work capacity, perhaps indicating a higher need of such information there.

While some previous studies state that SC guidelines have not improved clinical practice,[9 24] our findings suggest otherwise regarding the Swedish SC guidelines. Slightly more than half (55%) of the GPs in our study found that the guidelines facilitated their contacts with patients in sick-leave cases. Considering that our study included the responses from all GPs, and not

For peer review only - http://bmjopen.bmj.com/site/about/guidelines.xhtml

only those 90% who answered that they had used the SC guidelines as in Gustavsson's et al study,[14] the proportion who state that the guidelines facilitate their contacts with patients seem to have been fairly stable over time. In 2012, 61% of those who had used the SC guidelines reported that the guidelines facilitated their contacts with patients, and 56% reported this in 2008,[14] when the guidelines were newly implemented. Moreover, in analyses of ORs for stating that the guidelines facilitated their contacts with patients, among all sick-listing physicians, showed higher ORs among women (OR 1.23 (95% CI 1.15-1.32), younger physicians (OR aged 24-39: 3.13 (2-86-3.42); OR aged 40-54: 1.38 (1.26-1.51)), for non-specialists (OR 3.28 (3.04-3.55)), as well as for physicians in primary health care. Skånér et al[11] also found that non-specialists within primary healthcare to a larger extent stated that the guidelines facilitated their contacts with patients. Comparing results with regards to if SC guidelines are perceived to be of importance for the quality of the management of sickness certification, 13% of the GPs in our study found the guidelines not to be important at all, a higher proportion than the 9% Skånér et al found in 2012.[11]

Strength of the study are that nearly all physicians, not only a sample or a specific type of physicians (i.e., GPs), were invited, and the large number of respondents, allowing several sub-group analyses, e.g., types of clinics or level of education. Although the response rate (54%) was relatively high, a limitation is that we do not know if non-responders vary regarding the here studied questions. Nevertheless, that is improbable as the questions regarding guidelines came late in the comprehensive questionnaire of 133 questions. However, we might have an underestimation in rates of physicians stating they used the guidelines. As the guidelines had existed for ten years when answering the survey, some physicians who answer that they do not use guidelines, may state so because they are already familiar with the guidelines and the recommendations, and thus no longer actively check the web-site regarding, e.g., sick-leave duration or degree for a specific diagnosis. The fact that

For peer review only - http://bmjopen.bmj.com/site/about/guidelines.xhtml

the guidelines has become a part of many physicians daily sick-leave practice also means they may value them less highly than when they were just introduced ten years before, due to that the guidelines are not perceived to add any new information, as they previously might have done. The results of this study could be generalized to Sweden and probably other countries that have introduced SC guidelines, especially those with similar sickness absence and SC systems.

IMPLICATIONS FOR PRACTICE

Knowledge about use, usefulness, and problems regarding aspects of the SC guidelines is warranted to be able to improve their usefulness for different types of clinics. The finding that there were several types of clinics where a majority of physicians handled sick-leave cases every week but seldom used the guidelines, indicates that there might be a need for developing the guidelines, e.g., with regard to more diagnoses, or more useful and updated information. A clinical implication elucidated is that less experienced physicians, especially in clinical settings such as primary healthcare where sick-leave cases are common, should be provided information about the SC guidelines in workplace introductions.

CONCLUSIONS AND SUGGESTIONS FOR FURTHER RESEARCH

The lack of scientific knowledge concerning the use and relevance of SC guidelines is remarkable, given the considerable economic costs to patients, employers, healthcare, and society due to sick leave. Different types of clinics use different types of information in the guidelines, indicating varied needs. Further research should focus on factors that hinder and promote the use of SC guidelines and on what information physicians need from such guidelines, in general as well as in different types of clinics.

For peer review only - http://bmjopen.bmj.com/site/about/guidelines.xhtml

Contributors

KA initiated the project including the data collection, made the study design, developed the questionnaire, and is the guarantor. A statistician performed the data management and statistical analyses. VS drafted the manuscript and the tables. Both authors participated in presentation and interpretation of data and reviewed the manuscript.

Funding

The data collection was funded by the Swedish Social Insurance Agency (066754-2016). The Agency was not involved in the design of this study, analysis, interpretation of the data, nor as authors of the manuscript. The authors have had access to all data.

Competing interests

None.

Ethics approval Ethics approval for this project was granted by the Regional Ethical Review Board of Stockholm, Sweden (2017/95-32).

Data availability statement The data used in this study is administered by the Division of Insurance Medicine, Karolinska Institutet, and cannot be made publically. According to the General Data Protection Regulation, the Swedish law SFS 2018:218, the Swedish Data Protection Act, the Swedish Ethical Review Act, and the Public Access to Information and Secrecy Act, data can only be made available, after legal review, for researchers who meet the criteria for access to this type of confidential data. Readers may contact Professor Kristina Alexanderson (kristina.alexanderson@ki.se).

ORCID iDs

Veronica Svärd https://orcid.org/0000-0002-3868-0254

Kristina Alexanderson https://orcid.org/0000-0002-9313-3413

For peer review only - http://bmjopen.bmj.com/site/about/guidelines.xhtml

REFERENCES

1 The Swedish Social Insurance Agency. Social Insurance in Figures 2020. Availiable: https://www.forsakringskassan.se/wps/wcm/connect/cf56c741-1668-43c2-887d-10dd6c243cde/socialforsakringen-i-siffror-2020-engelsk.pdf?MOD=AJPERES&CVID= (Accessed 20 March 2021).

2 Söderberg E, Lindholm C, Kärrholm J, et al. Physician's sickness certification practices: A systematic review: Division of Insurance Medicine, Karolinska Institutet, Stockholm, 2019. ISBN 978-91-7831-362-4.

3 Ljungquist T, Hinas E, Nilsson G, et al. Problems with sickness certification tasks: experiences from physicians in different clinical settings. A cross-sectional nationwide study in Sweden. *BMC Health Serv Res* 2015;15:321.

4 Wynne-Jones G, Mallen C, Main C, et al. What do GPs feel about sickness certification? A systematic search and narrative review. *Scand J Prim Health Care* 2010;28:67-75.

5 Winde L, Alexanderson K, Carlsen B, et al. General practitioners' experiences with sickness certification: A comparison of survey data from Sweden and Norway. *BMC Fam Pract* 2012;13:10.

6 Wahlstrom R, Alexanderson K. Swedish Council on Technology Assessment in Health Care (SBU). Chapter 11. Physicians' sick-listing practices (review). *Scand J Public Health Suppl* 2004;63:222-55.

7 Hinkka K, Niemelä M, Autti-Rämö I, et al. Physicians' experiences with sickness absence certification in Finland. *Scand J Public Health* 2018:1403494818758817.

8 de Boer WE, Mousavi SM, Delclos GL, et al. Expectation of sickness absence duration: a review on statements and methods used in guidelines in Europe and North America. *Eur J Public Health* 2015;26(2):306-11.

9 Letrilliart L, Barrau A. Difficulties with the sickness certification process in general practice and possible solutions: a systematic review. *Eur J Gen Pract* 2012;18(4):219-28.

10 Reed P, editor. *The Medical Disability Advisor. Workplace Guidelines for Disability Duration*. Singapore: Reed Group Holdings, Ltd., 2004.

11 Skånér Y, Nilsson G, Arrelöv B, et al. Use and usefulness of guidelines for sickness certification: results from a national survey of all general practitioners in Sweden. *BMJ Open* 2011;Dec 20;1(2):e000303.

12 The Swedish Board of Health and Welfare. Försäkringsmedicinskt beslutsstöd - vägledning för sjukskrivning (reviderad 2012) (Sickness certification guidelines; revised 2012) (In Swedish), 2012. Availiable: https://roi.socialstyrelsen.se/fmb (Accessed 20 March 2021).

13 World Health Organization. International classification of functioning, disability and health (ICF), 2007. Available: https://www.who.int/standards/classifications/international-classification-of-functioning-disability-and-health (Accessed 20 March 2021).

14 Gustavsson C, Hinas E, Ljungquist T, et al. General practitioners' use of sickness certification guidelines in Sweden at introduction and four years later: a survey study. *Int J Qual Health Care* 2018;30(6):429-38.

15 Gustavsson C, Hinas E, Ljungquist T, et al. Obstetricians/Gynecologists' Problems in Sickness Certification Consultations: Two Nationwide Surveys. *Obstet Gynecol Int* 2016(Article ID 9421316):1-11.

16 Nilsing E, Söderberg E, Öberg B. Sickness certificates in Sweden: did the new guidelines improve their quality? *BMC Public Health* 2012;12(907).

17 Engblom M, Alexanderson K, Rudebeck CE. Physicians' messages in problematic sickness certification: a narrative analysis of case reports. *BMC Fam Pract* 2011;12:18.

18 Lindholm C, von Knorring M, Arrelöv B, et al. Health care management of sickness certification tasks: results from two survey to physicians. *BMC Res Notes* 2013;6(207).

19 Engblom M, Nilsson G, Arrelov B, et al. Frequency and severity of problems that general practitioners experience regarding sickness certification. *Scand J Prim Health Care* 2011;29(4):227-33.

For peer review only - http://bmjopen.bmj.com/site/about/guidelines.xhtml

20 Alexanderson K, Arrelöv B, Friberg E, et al. *Läkares erfarenheter av arbete med sjukskrivning av patienter. Resultat från en enkät år 2017 och jämförelser med resultat från motsvarande enkäter år 2012, 2008 respektive 2004. Huvudrapport 2018*. (Physician's experiences of handling sickness certification of patients. Results from 2017 questionnaire and comparisons with results from 2012, 2008, and 2004. Main report.) (In Swedish): Division of Insurance Medicine, Karolinska Institutet, 2018:1-265. ISBN 978-91-7676-054-3. Availiable: https://ki.se/sites/default/files/migrate/2018/03/08/huvudrapport_lakarenkat_2017_2012_2008_2004._ki.pdf (Accessed 21 March 2021).
21 Soderberg E, Alexanderson K. Sickness certification practices of physicians: a review of the literature. *Scand J Public Health* 2003;31(6):460-74.
22 Ljungquist T, Alexanderson K, Kjeldgård L, et al. Occupational health physicians have better work conditions for handling sickness certification compared with general practitioners: Results from a nationwide survey in Sweden. *Scand J Public Health* 2015;43(1):35-43.
23 Roope R, Parker G, Turner S. General practitioners' use of sickness certificates. *Occup Med* 2009;59(8):580-5.
24 Nordhagen H, Harvey S, Rosvold E, et al. Case-specific colleague guidance for general practitioners' management of sickness absence. *Occup Med* 2017;67(8):644-47.

For peer review only

For peer review only - http://bmjopen.bmj.com/site/about/guidelines.xhtml

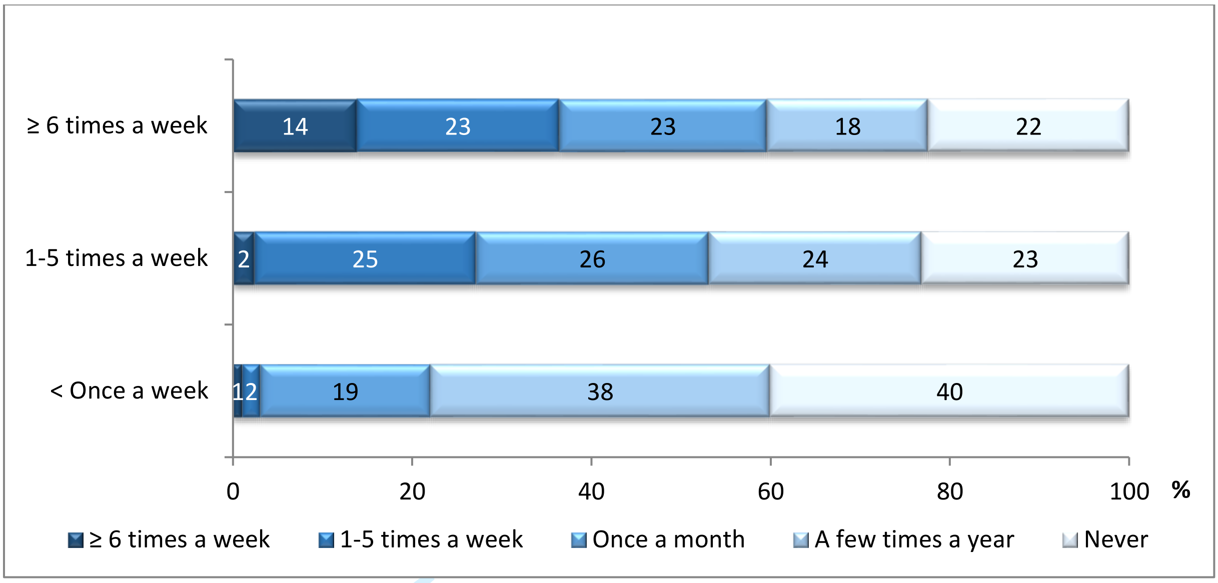

Figure 1. Proportion (%) of physicians who used the sickness certification guidelines with different frequencies (X-axis), by how often they had sick-leave cases (Y-axis).

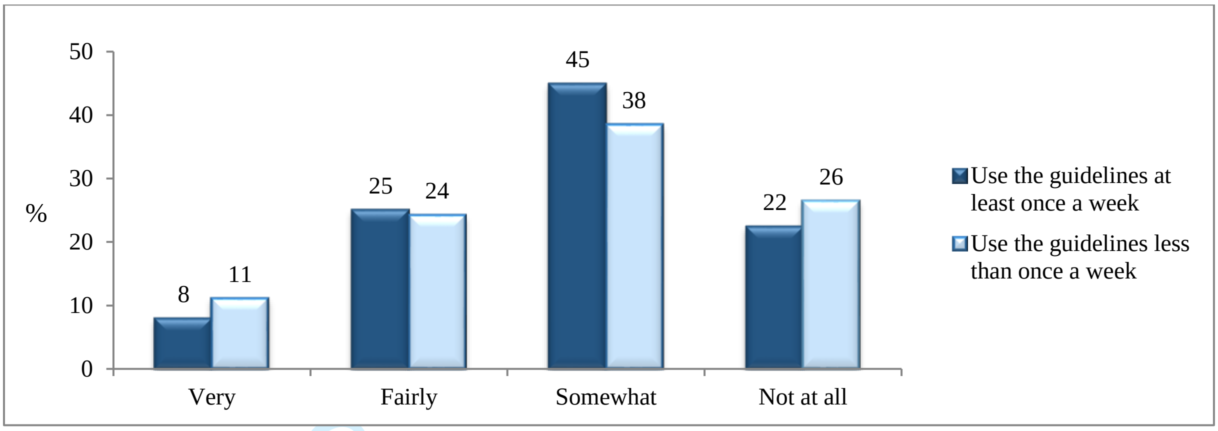

Figure 2. Proportions (%) of physicians who stated it was problematic to apply the sickness certification guidelines, by how often they used the guidelines.

STROBE 2007 (v4) Statement—Checklist of items that should be included in reports of *cross-sectional studies*

Section/Topic	Item #	Recommendation	Reported on page #
**Title and abstract**	1	(*a*) Indicate the study's design with a commonly used term in the title or the abstract	2
		(*b*) Provide in the abstract an informative and balanced summary of what was done and what was found	2
**Introduction**			
Background/rationale	2	Explain the scientific background and rationale for the investigation being reported	4-6
Objectives	3	State specific objectives, including any prespecified hypotheses	6
**Methods**			
Study design	4	Present key elements of study design early in the paper	6
Setting	5	Describe the setting, locations, and relevant dates, including periods of recruitment, exposure, follow-up, and data collection	6-7
Participants	6	(*a*) Give the eligibility criteria, and the sources and methods of selection of participants	7
Variables	7	Clearly define all outcomes, exposures, predictors, potential confounders, and effect modifiers. Give diagnostic criteria, if applicable	8
Data sources/ measurement	8*	For each variable of interest, give sources of data and details of methods of assessment (measurement). Describe comparability of assessment methods if there is more than one group	8
Bias	9	Describe any efforts to address potential sources of bias	*7*
Study size	10	Explain how the study size was arrived at	8
Quantitative variables	11	Explain how quantitative variables were handled in the analyses. If applicable, describe which groupings were chosen and why	8
Statistical methods	12	(*a*) Describe all statistical methods, including those used to control for confounding	8
		(*b*) Describe any methods used to examine subgroups and interactions	8
		(*c*) Explain how missing data were addressed	8
		(*d*) If applicable, describe analytical methods taking account of sampling strategy	N.A.
		(*e*) Describe any sensitivity analyses	N.A.
**Results**			

For peer review only

Participants	13*	(a) Report numbers of individuals at each stage of study—eg numbers potentially eligible, examined for eligibility, confirmed eligible, included in the study, completing follow-up, and analysed	8-9
		(b) Give reasons for non-participation at each stage	7
		(c) Consider use of a flow diagram	N.A.
Descriptive data	14*	(a) Give characteristics of study participants (eg demographic, clinical, social) and information on exposures and potential confounders	8
		(b) Indicate number of participants with missing data for each variable of interest	8
Outcome data	15*	Report numbers of outcome events or summary measures	7-8
Main results	16	(*a*) Give unadjusted estimates and, if applicable, confounder-adjusted estimates and their precision (eg, 95% confidence interval). Make clear which confounders were adjusted for and why they were included	14
		(*b*) Report category boundaries when continuous variables were categorized	N.A.
		(*c*) If relevant, consider translating estimates of relative risk into absolute risk for a meaningful time period	Not relevant
Other analyses	17	Report other analyses done—eg analyses of subgroups and interactions, and sensitivity analyses	N.A.
**Discussion**			
Key results	18	Summarise key results with reference to study objectives	14
Limitations	19	Discuss limitations of the study, taking into account sources of potential bias or imprecision. Discuss both direction and magnitude of any potential bias	16-17
Interpretation	20	Give a cautious overall interpretation of results considering objectives, limitations, multiplicity of analyses, results from similar studies, and other relevant evidence	16-17
Generalisability	21	Discuss the generalisability (external validity) of the study results	17
**Other information**			
Funding	22	Give the source of funding and the role of the funders for the present study and, if applicable, for the original study on which the present article is based	18

*Give information separately for cases and controls in case-control studies and, if applicable, for exposed and unexposed groups in cohort and cross-sectional studies.

Note: An Explanation and Elaboration article discusses each checklist item and gives methodological background and published examples of transparent reporting. The STROBE checklist is best used in conjunction with this article (freely available on the Web sites of PLoS Medicine at http://www.plosmedicine.org/, Annals of Internal Medicine at http://www.annals.org/, and Epidemiology at http://www.epidem.com/). Information on the STROBE Initiative is available at www.strobe-statement.org.

For peer review only

Physician’s use of sickness certification guidelines: a nationwide survey of 13 750 physicians in different types of clinics in Sweden

Journal:	*BMJ Open*
Manuscript ID	bmjopen-2021-051555.R1
Article Type:	Original research
Date Submitted by the Author:	20-Nov-2021
Complete List of Authors:	Svärd, Veronica; Karolinska Institutet, Division of Insurance Medicine, Department of Clinical Neuroscience; Södertörn University, Department of Social Work<br>Alexanderson, Kristina; karolinska institutet, Department of Clinical Neuroscience
**Primary Subject Heading**:	Public health
Secondary Subject Heading:	Occupational and environmental medicine, Rehabilitation medicine, Health policy
Keywords:	Health policy < HEALTH SERVICES ADMINISTRATION & MANAGEMENT, PUBLIC HEALTH, SOCIAL MEDICINE, Protocols & guidelines < HEALTH SERVICES ADMINISTRATION & MANAGEMENT

I, the Submitting Author has the right to grant and does grant on behalf of all authors of the Work (as defined in the below author licence), an exclusive licence and/or a non-exclusive licence for contributions from authors who are: i) UK Crown employees; ii) where BMJ has agreed a CC-BY licence shall apply, and/or iii) in accordance with the terms applicable for US Federal Government officers or employees acting as part of their official duties; on a worldwide, perpetual, irrevocable, royalty-free basis to BMJ Publishing Group Ltd ("BMJ") its licensees and where the relevant Journal is co-owned by BMJ to the co-owners of the Journal, to publish the Work in this journal and any other BMJ products and to exploit all rights, as set out in our licence.

The Submitting Author accepts and understands that any supply made under these terms is made by BMJ to the Submitting Author unless you are acting as an employee on behalf of your employer or a postgraduate student of an affiliated institution which is paying any applicable article publishing charge ("APC") for Open Access articles. Where the Submitting Author wishes to make the Work available on an Open Access basis (and intends to pay the relevant APC), the terms of reuse of such Open Access shall be governed by a Creative Commons licence – details of these licences and which Creative Commons licence will apply to this Work are set out in our licence referred to above.

Other than as permitted in any relevant BMJ Author's Self Archiving Policies, I confirm this Work has not been accepted for publication elsewhere, is not being considered for publication elsewhere and does not duplicate material already published. I confirm all authors consent to publication of this Work and authorise the granting of this licence.

For peer review only

Physician's use of sickness certification guidelines: a nationwide survey of 13 750 physicians in different types of clinics in Sweden

Veronica Svärd & Kristina Alexanderson

The Division of Insurance Medicine, Department of Clinical Neuroscience, Karolinska Institutet, SE-171 77 Stockholm, Sweden

Corresponding author: Veronica Svärd, veronica.svard@ki.se

Word count: 3641

For peer review only

For peer review only - http://bmjopen.bmj.com/site/about/guidelines.xhtml

ABSTRACT

Objectives: To explore physicians' experiences of using the national sickness certification guidelines introduced in 2007 and the types of information they used, in general and in different types of clinics.

Design: Cross-sectional survey.

Setting: Most physicians working in Sweden in 2017.

Participants: A questionnaire was sent to 34 718 physicians; 54% responded. Analyses were based on answers from the 13 750 physicians who had sick-leave cases.

Outcome measures: To what extent the guidelines were used and what type of information from them that was used.

Results: Ten years after the sickness certification guidelines were introduced in Sweden, half of the physicians used them at least once a month. About 40% of physicians in primary healthcare and occupational health services used the guidelines every week. The type of information used varied; 53% used recommendations about duration and 29% about degree of sick leave. Using information about function and activity/work capacity, respectively, was more common within primary healthcare (37% and 38%), psychiatry (42% and 42%), and occupational health services (35% and 41%), and less common in surgery and orthopedic clinics (12% and 12%) who more often used information about duration (48% and 53%). Moreover, 10% stated that the guidelines were very, and 24% fairly problematic to apply. Half (47%) stated that the guidelines facilitated their contacts with patients and 29% that they improved quality in their management of sick-leave cases. More non-specialists, compared to specialists, found that the guidelines facilitated contacts with patients (odds ratio: 3.28 (95% confidence interval: 3.04-3.55)).

For peer review only - http://bmjopen.bmj.com/site/about/guidelines.xhtml

Conclusions: The majority of the physicians used the sickness certification guidelines, although this varied with type of clinic. Half stated that the guidelines facilitated patient contacts. Yet, some found it problematic to apply the guidelines. Further development of the guidelines is warranted as well as more knowledge about them among physicians.

Keywords: sick leave, sickness certification, physician, guidelines, clinics

For peer review only

STRENGTHS AND LIMITATIONS OF THIS STUDY

- With 13 750 responding sick-listing physicians this is the largest survey of physicians' work with sick-leave cases, also internationally, allowing several types of subgroup analyses.
- All physicians involved in sickness certification were included, not only GPs, which is seldom the case in this research area.
- Although the response rate (54%) was relatively high for this type of study, we do not know if non-responders would have responded differently.
- Comparisons with findings from the few studies from other countries is problematic due to large differences in both sickness absence insurance systems and in available sickness certification guidelines.
- The cross-sectional design means that no conclusions about causality can be drawn.

For peer review only - http://bmjopen.bmj.com/site/about/guidelines.xhtml

INTRODUCTION

In Sweden, the sick-leave rates have fluctuated much in the last decades,[1] and many interventions have been initiated to keep the rates at a more stable and lower level. Several of the interventions have been directed towards physicians, who have a central role in the sick-leave process. Systematic reviews have found that physician's find aspects of sickness certification (SC) of patients as problematic,[2-6] e.g., the following aspects: their own relative lack of competence regarding insurance medicine, lack of support for handling sickness certification, and the general lack of scientific knowledge regarding optimal sick leave and handling of such work tasks.[2-6] This is one of the reasons for why different types of SC guidelines have been asked for and, in some countries also introduced.[7-11] In Sweden, such guidelines were introduced in 2007.

SC guidelines are to assist clinicians in their handling of consultations regarding sickness absence. These types of guidelines serve, when they exist, as recommendations for clinicians to uphold the best practice regarding SC. Some of the guidelines that have been introduced are, however, merely based on data regarding duration of previous sick-leave cases,[10] which involves several problems when put in practical use. An essential barrier to develop guidelines in Sweden as well as in other countries, is the lack of scientific knowledge regarding the need for, as well as possible consequences/side effects of being on sick leave for different periods of time among patients with specific medical conditions. Moreover, such knowledge would be needed in relation to different types of work task the patient has, as it is not a diagnosis in itself that gives the right to be on sick leave but the extent to which the diagnosis limits functions that are of importance for that individual's work capacity – that is, in relation to his/her specific work tasks.

Due to the limited number of studies regarding optimal and/or needed sick leave among people with different medical conditions and work tasks, such guidelines so far cannot be

For peer review only

For peer review only - http://bmjopen.bmj.com/site/about/guidelines.xhtml

based on scientific evidence.[8] Although Letrilliart and Barraur in a systematic review about issues regarding SC, argued that one strategy to improve sick-listing practice would be to provide guidelines, they found that general practitioners (GP) in Europe demonstrate low awareness and use of SC guidelines, and that the guidelines are ineffective without training in how to use them.[9]

Most studies regarding physicians' SC practice have so far focused on GPs.[2 4-6] However, in Sweden, as in many countries, all physicians, not only GPs can issue sick-leave certificates. In Sweden, such a certificate is needed after the 7th day of a sick-leave spell. The sickness certificate is used by the officers of the Social Insurance Agency to assess whether the patient/claimant fulfills the criteria for sick-leave benefits, which can be granted for full- or part-time (100, 75, 50, or 25%) of ordinary work hours. However, as clear from above, there is a nearly total lack of scientific evidence on optimal sick-leave duration and degree for patients with specific diagnoses and work tasks. Therefore, the SC guidelines for specific diagnoses were developed in Sweden by the National Board of Health and Welfare, based on discussions in expert groups, rather than based on established scientific evidence. For many diagnoses and for less severe conditions, the recommendation in the guidelines was no sick leave at all, or just for a few days or weeks. For other diagnoses, longer sick-leave spells could be accepted, especially if the patient had a physically demanding job. The Swedish National Board of Health and Welfare issued the web-based nationwide SC guidelines in 2007. They consist of two sets of recommendations: general guidelines covering the principles related to sickness certification and the diagnosis-specific recommendations.[12] The latter includes recommendations for duration and degree (full- or part-time) of sick leave as well as diagnosis-specific information about assessments of function, activity, and work capacity.[13] They cover about 110 of the most prevalent sick-leave diagnoses and are continuously updated, based on experiences from physicians and others, inconsistencies or

For peer review only

For peer review only - http://bmjopen.bmj.com/site/about/guidelines.xhtml

ambiguities in the texts, effects on work capacity from new treatments, continuous expert discussions, etcetera.

Previous studies about physician's use of these SC guidelines in Sweden show that a large majority (>85%) of GPs who have used guidelines find them beneficial to ensure high quality in handling sick-leave cases.[14] The use of guidelines among GPs[14] and among gynecologists/obstetricians[15] have increased over the years, as did the rate of gynecologists/obstetricians who reported that the guidelines facilitated their contacts with patients.[15] Another study compared the quality of sickness certificates before and after the SC guidelines were introduced, in 2007 respectively 2009.[16] Improvement of the quality of the information provided in the certificates was found regarding descriptions on functioning - although body impairments still dominated the descriptions of functioning, contradictory to the SC guidelines emphasizing of that functioning should be described in terms of activity and work incapacity. These previous studies do not, however, provide any knowledge about what type of information from the guidelines physicians find useful, and if this differs between type of clinic, such as psychiatry, orthopedics, and primary healthcare. Therefore, the aim of this study was to explore physicians' experiences of using the nationwide SC guidelines and what kind of information in the guidelines they used, and how this differed with type of clinic.

METHODS

Analyses of answers to a questionnaire sent to 34 718 physicians working and living in Sweden.

The questionnaire

A questionnaire about physician's work with sick-leave consultation including 133 questions was developed, based on previous surveys (2004, 2008, and 2012).[17-20] The items in the previous questionnaires were based on results from interviews, pilot studies, and other

For peer review only - http://bmjopen.bmj.com/site/about/guidelines.xhtml

studies.[2 21] Minor revisions of the 2012 questionnaire were made, based on previous results and extensive discussions with physicians and with representatives from physician organizations and other stakeholders.

The questionnaire included questions about frequency of sick-leave consultations, frequencies and severity of experienced problems, organizational prerequisites, internal and external cooperation, competence in insurance medicine, and about the use of the Swedish SC guidelines – the latter was the focus of this study.

Participants

The questionnaire was sent to all the physicians, 68 years and younger, living and working in Sweden in 2017, identified from the Swedish Healthcare Address Register of all physicians working in Sweden, administrated by QuintilesIMS. Specialists working in clinics that seldom have sick-leave cases were excluded, e.g., in laboratory, radiology, forensic, geriatric, and pediatric clinics. Statistics Sweden administrated the data collection, to ensure the respondents anonymous participation, and delivered an anonymized data file to the project group, including information on sex and age.

The questionnaire and a prepaid response envelope were sent to the participant's home addresses together with a cover letter with information about the project and that participation was voluntary. The questionnaire could be answered either over the web or by paper. Four reminders were sent to those who had not yet responded. The participants provided consent by submitting the filled questionnaire. It took about 30 minutes to fill out.

The study population included 34 718 physicians, of which 34 585 were eligible, and 18 714 of them responded (54.1% response rate). As usual in surveys, the response rate was somewhat higher among women (56.9% among women and 51.6% among men) and older physicians (51.9% among 20-54 years old and 59.2% among 55-67 years old).

For peer review only - http://bmjopen.bmj.com/site/about/guidelines.xhtml

Patient and public involvement

There were no patients involved in this study.

Statistical analysis

Descriptive statistics were used. Variables about the use of the SC guidelines were analyzed and comparisons were made between types of clinics regarding the following items: educational level, type of clinic, frequency of sick-leave cases, use of the guidelines, and if the guidelines were experienced as useful. Further variables concerned what information in the guidelines that were used: recommendations about duration and degree, information about function, activity/work capacity, or none. Logistic regression was used to calculate crude and adjusted odds ratios (OR) with 95% confidence intervals (CI) regarding if the physicians experienced that the SC guidelines facilitated their contacts with patients/make such contacts easier. Adjustments were conducted for being a specialist and for having sick-leave cases at least once a week.

In total, 13 750 physicians (78% of the respondents) had sick-leave cases at least once a year and those were included in the study. Those not specifying level of education (0.2% of all) or main type of clinic (0.3%, n=46) were not included in analyses related to level of education and type of clinic, respectively. For the specific questions about the SC guidelines, answers were missing for 2.2 – 4.9% of respondents and for one question 7.3%. Those were not included in the analyses of the respective question.

RESULTS

Of the sick-listing physicians, half (50.4%; n=6929) were women and 68% were board-certified specialists. A third (34%) worked in primary healthcare (Table 1), where the proportion of board-certified specialists was somewhat lower (61%). The highest proportions of physicians having sick-leave cases at least six times a week were in falling order in

For peer review only - http://bmjopen.bmj.com/site/about/guidelines.xhtml

orthopedics (74%), occupational health services (72%), rehabilitation (69%), oncology (61%), psychiatry (58%), pain (54%), and primary healthcare (45%).

Half (50%) used the SC guidelines at least once a month and 26% at least once a week. The frequency in use varied with type of clinic; physicians in occupational health services used the guidelines most often; 18% at least six times a week and 42% at least once a week. This was expected as all their patients are of working age and employed. About the same proportion of the GPs (41%) used the guidelines at least once a week.

Those who had sick-leave cases often also used the guidelines more often (Figure 1). The proportion (22-23%) who did not use the guidelines were the same irrespective if the frequency of sick-leave cases were 1-5 times a week or more than 6 times a week. Among those who had such consultations less than once/week, 40% stated that they did not use the guidelines.

For peer review only

For peer review only - http://bmjopen.bmj.com/site/about/guidelines.xhtml

Table 1. Number and proportion (%) of sick-listing physicians, proportions (%) of frequency of sick-leave cases, of frequency in use, and of types of information in the sickness certification guidelines used, by type of clinic (falling order according to size).

			Frequency of sick-leave cases			Frequency in using the guidelines					Type of information used from the guidelines					
Type of clinic	n	%[1]	≥6 cases a week	1-5 cases a week	Monthly	At least 6 times a week	1-5 times a week	Monthly	A few times a year	Never /almost never	None	Duration	Part- /full-time	Function	Activity /work capacity	Other
Primary healthcare	4687	34.1	45	50	4	9	32	31	18	10	18	69	43	37	38	3
Internal medicine	1661	12.1	12	46	41	2	10	22	34	32	37	53	28	19	18	2
Surgery	1285	9.3	27	52	21	4	13	18	27	38	46	48	18	12	12	1
Psychiatry	1050	7.6	58	32	10	11	23	27	20	19	28	45	29	42	42	3
Other	1029	7.5	33	20	36	4	11	18	24	43	44	37	20	16	17	1
Orthopedic	921	6.7	74	23	3	11	21	20	21	27	39	53	15	12	12	2
Ear, nose and throat	421	3.1	9	56	35	0	3	13	30	53	52	38	17	14	14	2
Infection	357	2.6	9	55	36	1	7	18	34	41	44	50	20	10	10	0
Oncology	342	2.5	61	31	8	5	11	20	25	39	50	34	13	16	15	2
Eye	341	2.5	3	19	78	1	3	8	28	60	67	25	9	8	9	1
Occupational health services	338	2.5	72	20	8	18	24	22	22	13	22	59	36	35	41	4
Gynecology	320	2.3	21	51	28	5	19	22	26	27	37	45	33	18	21	1
Neurology	305	2.2	29	58	13	1	12	16	35	36	46	38	23	20	19	2
Rheumatology	198	1.4	24	61	16	2	13	17	31	37	46	36	23	23	24	3
Skin	171	1.2	4	8	88	1	2	6	37	54	59	35	15	11	12	1
Rehabilitation	158	1.1	69	23	7	10	14	15	35	25	40	40	18	23	25	3
Administration[2]	66	0.5	9	30	60	3	10	13	25	49	45	39	24	18	26	2
Pain	54	<0.5	54	33	13	10	4	14	24	47	54	22	17	24	19	4
**All physicians**	**13 750**	**100**	**38**	**43**	**19**	**6**	**20**	**24**	**24**	**26**	**33**	**53**	**29**	**25**	**26**	**2**

[1]Column percentage, all other row percentages.

[2]Including research and education (i.e., physicians mainly working with education, research, or as managers but also having patients some of the time).

For peer review only - http://bmjopen.bmj.com/site/about/guidelines.xhtml

Regarding what type of information from the guidelines the physicians used, more than half (53%) used recommendations about duration of sick leave, while 29% used recommendations about degree (part- or full-time sick leave) (Table 1). Half or more of the physicians within primary healthcare (69%), occupational health services (59%), internal medicine (53%), orthopedics (53%), and infection (50%) used the recommendations about duration. Using recommendations about degree was most common in primary healthcare (43%), gynecology (33%), and occupational health services (36%). Use of the information about limitation of function or about activity limitation/work capacity was more common within primary healthcare (37% and 38%, respectively), psychiatry (42% and 42%), and occupational health services (35% and 41%). A majority of the physicians within eye (67%), skin (59%), oncology (50%), pain (54%), and ear, nose, and throat clinics (52%) did not use any information from the guidelines, partly due to lack of SC guidelines regarding several diagnoses handled in those clinics. Also, the proportions varied *within* types of clinics regarding type of information used. While about half of the physicians within surgery and orthopedics used information about duration, only 12% used information about limitation of function or activity/work capacity (Table 1). Within psychiatry, on the other hand, almost the same proportions used information about limitation of function or activity/work capacity (42%) as recommendations about the duration (45%).

Ten percent of those who used the guidelines stated that the they were very problematic to apply and 24% that they were fairly problematic to apply, while 25% never found them problematic to apply. A somewhat higher proportion of those who used the guidelines less than once a week (11%) compared to at least once a week (8%) stated that they were very problematic to use (Figure 2).

Nearly a third (29%) reported that the guidelines to a high extent improved the quality in their management of SC; and higher proportions in gynecology (35%) and internal medicine (33%)

For peer review only - http://bmjopen.bmj.com/site/about/guidelines.xhtml

stated this, compared to, e.g., in the clinics with fewer physicians such as rehabilitation (17%) and pain clinics (19%) (Table 2).

Table 2. Proportion (%) of physicians who stated that the sickness certification guidelines are important for good quality in her/his handling of sickness certification and facilitate their contacts with patients or others, by type of clinic.

	The extent to which guidelines is of importance for the quality of how I handle sickness certification			The guidelines facilitate my contacts with ...					
Type of clinic	Very	Moderately	None	The patient	Social Insurance Agency	Health care staff	Employer	Employ-ment Office	At least one of all
Primary healthcare	31	57	13	55	45	33	23	25	65
Internal medicine	33	51	16	41	41	31	22	22	53
Surgery	30	49	21	46	34	28	18	17	48
Psychiatry	30	54	16	40	43	31	22	25	55
Other	30	45	25	35	35	30	19	20	39
Orthopedic	24	51	25	44	35	24	16	16	50
Ear, nose and throat	23	56	22	39	31	22	17	18	41
Infection	32	54	15	44	35	23	18	16	46
Oncology	24	55	21	35	37	20	13	13	42
Eye	21	56	23	26	25	25	13	14	27
Occupational health services	22	62	16	46	37	24	31	17	58
Gynecology	35	51	15	48	37	29	19	16	51
Neurology	21	59	20	31	32	17	12	14	42
Rheumatology	21	49	30	34	33	24	19	19	41
Skin	26	54	20	40	27	24	15	16	40
Rehabilitation	17	63	20	32	30	22	19	19	42
Administration	21	55	23	33	36	18	18	16	45
Pain	19	57	31	33	28	28	18	20	33
**All physicians**	**29**	**54**	**17**	**47**	**39**	**29**	**21**	**21**	**53**

Half (53%) stated that the guidelines facilitated their contacts, or made the contacts easier, with at least one of the suggest; i.e., patient, Social Insurance Agency, healthcare staff, patients' employer, and/or employment office. About twice as high proportions in primary healthcare (65%) and occupational health services (58%) stated this compared to among the physicians in smaller clinics that seldom used the guidelines, such as eye clinics (27%). Lower proportions stated that the guidelines facilitated their contacts with employers or

For peer review only - http://bmjopen.bmj.com/site/about/guidelines.xhtml

employment offices (21%) and healthcare staff (29%) compared to with the Social Insurance Agency (39%) and patients (47%). However, this varied with type of clinic. Here it is important to remember that all physicians had contacts with their patients, while only a few had contacts with the other stakeholders, which is why the figures for those are expected to be lower. A third (31%) of physicians in occupational health services stated that the guidelines facilitated their contacts with patients' workplaces. Among those mainly working in oncology (37%), psychiatry (43%), or neurology (32%), the highest proportions stated that the guidelines facilitated their contacts with the Social Insurance Agency. For most type of clinics, however, the highest proportions reported that the guidelines facilitated their contacts with the patient.

The ORs for stating that the SC guidelines facilitated the contact with the patient was higher among women (OR 1.23 (95% CI 1.15-1.32) then men, among younger physicians (OR aged 24-39: 3.13 (2-86-3.42); OR aged 40-54: 1.38 (1.26-1.51)), compared to the older physicians (aged 55-68). Also, the OR regarding this was more than three times as high for non-specialists (OR 3.28 (3.04-3.55)), compared with specialists. As shown in Table 2, a higher rate of the physicians in primary healthcare stated that the guidelines facilitated contacts with patients (55%). In Table 3, the ORs for stating this is shown for five specific clinical settings, using the 4477 Other physicians as reference group. For all types of clinics, ORs are statistically significant, with the exception of psychiatry, also when adjusting for being a specialist and for having sick-leave cases at least once a week. The GP's, OR was especially high (adjusted OR 1.78 (1.62-1.95). When restricting those analyses to the physicians who at least once a month used the guidelines, the results differed; after adjustments the OR in the group Other physicians (n=1415) was somewhat higher than in several of the other clinics, however, only significantly so for those in psychiatric clinics.

For peer review only - http://bmjopen.bmj.com/site/about/guidelines.xhtml

Table 3. Crude and adjusted odds ratio (OR) with 95% confidence intervals (CI) for stating that the sickness certification guidelines facilitate the communication with patients, in different type of clinics. Among all physicians and among physicians who at least once a month used the guidelines.

Type of clinic	Crude OR	95% CI	Adjusted OR[1]	95% CI	Adjusted OR[2]	95% CI	Adjusted OR[3]	95% CI
All								
All other physicians (n=4477)	Ref		Ref		Ref		Ref	
Internal medicine	1.51	1.35-1.70	1.46	1.30-1.65	1.49	1.32-1.67	1.43	1.27-1.62
Psychiatry	1.02	0.89-1.97	1.03	0.89-1.19	1.05	0.91-1.21	1.07	0.92-1.24
Orthopedic	1.17	1.01-1.17	1.25	1.07-1.45	1.22	1.05-1.41	1.31	1.12-1.53
Occupational health services	1.30	1.04-1.35	1.82	1.45-2.28	1.22	1.05-1.41	1.90	1.51-2.39
Primary healthcare	1.81	1.66-1.63	1.70	1.56-1.85	1.88	1.72-2.05	1.78	1.62-1.95
Used the guidelines at least monthly								
All other physicians (n=1415)	Ref		Ref		Ref		Ref	
Internal medicine	1.29	1.04-1.60	1.23	0.98-1.53	1.17	0.94-1.46	1.12	0.90-1.41
Psychiatry	0.53	0.44-0.65	0.56	0.46-0.68	0.55	0.46-0.67	0.58	0.48-0.71
Orthopedic	0.93	0.74-1.16	1.00	0.80-1.26	0.99	0.79-1.24	1.07	0.85-1.34
Occupational health services	0.67	0.50-0.89	0.90	0.67-1.21	0.71	0.53-0.95	0.96	0.71-1.29
Primary healthcare	0.93	0.81-1.05	0.94	0.82-1.07	0.98	0.86-1.12	0.99	0.86-1.13

[1] Adjusted for specialists.

[2] Adjusted for having sick-leave cases at least once a week.

[3] Adjusted for both [1] and [2].

For peer review only

DISCUSSION

In this study, a very large number (13 750) of the sick-listing physicians in Sweden responded to questions about their use of the SC guidelines, ten years after the guidelines were introduced in Sweden in 2007. Half (50%) used the guidelines at least once a month and 26% at least once a week, although those rates differed much with type of clinic and also regarding what type of information the physician used. Half (47%) stated that the guidelines facilitated their contacts with patients regarding SC and discussion about sick leave. Furthermore, 29% of the physicians stated that the guidelines improved quality in their management of SC cases. Ten percent stated that the guidelines were very problematic to apply and 17% did not experienced that the guidelines improved the quality of their handling of SC.

Comparing results with those from previous Swedish studies shows that the every-week use increased among GPs from 22% in 2008[11] and 29% in 2012[14] to 41% in 2017, that is, in this survey. Among occupational health physicians, every week use increased from 33% in 2008[22] to 42% in 2017. Internationally, there are hardly no equivalent studies, however, in the UK it was found that four years after introduction of SC guidelines, 36% of the 77 responding GPs were aware of the guidelines and 20% of these 77 GPs used them.[23] This might be compared to the 76% of the responding GPs who used guidelines at least a few times a year already one year after the guidelines were introduced in Sweden in 2007.[11] While Letrilliart and Barraurs systematic review found that GPs in Europe demonstrate low awareness and use of SC guidelines,[9] our findings show that this is not the case in Sweden. However, precise comparisons cannot be made between the studies, conducted in different European countries, because of different selections, contexts, and regulations; yet they show that differences between the countries exists. One reason for the high awareness and use of the SC guidelines in Sweden might be that the Social Insurance Agency use the guidelines when assessing claimant's right to sick-leave benefits. The relatively high use among physicians in

For peer review only - http://bmjopen.bmj.com/site/about/guidelines.xhtml

occupational health services should be related to that they only have employed patients, while other physician to a larger extent have patients who not are in paid work, or not even of working age.

For some groups of diagnoses there are fewer diagnosis-specific SC guidelines than for other groups, which probably explains some of the large variations between different type of clinics' use of the guidelines. Another finding was that the type of information used varied somewhat with type of clinic. In psychiatry, a comparatively high proportion used information about function and activity/work capacity, perhaps meaning these guidelines were particularly well written, or that psychiatrists need such information more than other physicians. In other type of clinics, such as internal medicine, neurology, gynecology, and pain, information about function limitations were used to a higher extent than information about activity/work capacity, perhaps indicating a higher need of such information there.

While some previous studies state that SC guidelines have not improved clinical practice,[9 24] our findings suggest otherwise regarding the Swedish SC guidelines. Slightly more than half (55%) of the GPs in our study found that the guidelines facilitated their contacts with patients in sick-leave cases. Considering that our study included the responses from all participating GPs, and not only those 90% who answered that they had used the SC guidelines as in the study by Gustavsson et al,[14] the proportion who stated that the guidelines facilitate their contacts with patients seem to have been fairly stable over time. In 2012, 61% of those who had used the SC guidelines reported that the guidelines facilitated their contacts with patients, and 56% reported this in 2008,[14] when the guidelines were newly implemented. Moreover, in analyses of ORs for stating that the guidelines facilitated their contacts with patients, among all sick-listing physicians, showed higher ORs among women (OR 1.23 (95% CI 1.15-1.32), younger physicians (OR aged 24-39: 3.13 (2-86-3.42); OR aged 40-54: 1.38 (1.26-1.51)), as well as for non-specialists (OR 3.28 (3.04-3.55)). This is in line with results from Skånér et

For peer review only - http://bmjopen.bmj.com/site/about/guidelines.xhtml

al[11] that non-specialists within primary healthcare to a larger extent stated that the guidelines facilitated their contacts with patients. Moreover, the adjusted ORs for experiencing that the SC guidelines facilitated the physician's contacts with patients was highest among GPs. Comparing results with regards to if SC guidelines are perceived to be of importance for the quality of the management of sickness certification, 13% of the GPs in our study found the guidelines not to be important at all, a higher proportion than the 9% Skånér et al found in 2012.[11]

Strength of the study are that nearly all physicians, not only a sample or a specific type of physicians (i.e., GPs), were invited, and the large number of respondents, allowing several sub-group analyses, e.g., types of clinics or level of education. Although the response rate (54%) was relatively high, a limitation is that we do not know if non-responders vary regarding the here studied questions. Nevertheless, that is improbable as the questions regarding guidelines came late in the comprehensive questionnaire of 133 questions. It is possible that physicians who experience themselves being too busy to answer the questionnaire, also experience themselves being too busy to search for and use the SC guidelines. However, we might also have an underestimation in rates of physicians stating they used the guidelines. As the guidelines had existed for ten years when answering the survey, some physicians who answer that they do not use guidelines, may state so because they are already familiar with the guidelines and the recommendations, and thus no longer actively check the web-site regarding, e.g., sick-leave duration or degree for a specific diagnosis. The fact that the guidelines has become a part of many physicians daily SC practice also means they may value them less highly than when they were introduced ten years before, due to that the guidelines are not perceived to add any new information, in the way they previously might have done. The results of this study could be generalized to Sweden and

For peer review only - http://bmjopen.bmj.com/site/about/guidelines.xhtml

probably other countries that have introduced these types of SC guidelines, especially those with similar sickness absence and SC systems.

IMPLICATIONS FOR PRACTICE

Knowledge about use, usefulness, and problems regarding aspects of the SC guidelines is warranted to be able to improve their usefulness for different types of clinics. The finding that there were several types of clinics where a majority of physicians handled sick-leave cases every week but seldom used the guidelines, indicates that there might be a need for developing the guidelines, e.g., with regard to more diagnoses, or more useful and updated information. A clinical implication elucidated is that less experienced physicians, especially in clinical settings such as primary healthcare where sick-leave cases are common, should be provided information about the SC guidelines in workplace introductions.

CONCLUSIONS AND SUGGESTIONS FOR FURTHER RESEARCH

The lack of scientific knowledge concerning the use and relevance of SC guidelines is remarkable, given the considerable economic costs to patients, employers, healthcare, and society due to sick leave. Physicians in different types of clinics use different types of information in the guidelines, indicating varied needs. Further research should focus on factors that hinder and promote the use of SC guidelines and on what information physicians need from such guidelines, in general as well as in different types of clinics.

Contributors

KA initiated the project including the data collection, made the study design, developed the questionnaire, and is the guarantor. A statistician performed the data management and statistical analyses. VS drafted the manuscript and the tables. Both authors participated in presentation and interpretation of data and reviewed the manuscript.

For peer review only - http://bmjopen.bmj.com/site/about/guidelines.xhtml

Funding

The data collection was funded by the Swedish Social Insurance Agency (066754-2016). The Agency was not involved in the design of this study, analysis, interpretation of the data, nor as authors of the manuscript. The authors have had access to all data.

Competing interests

None.

Ethics approval Ethics approval for this project was granted by the Regional Ethical Review Board of Stockholm, Sweden (2017/95-32).

Data availability statement The data used in this study is administered by the Division of Insurance Medicine, Karolinska Institutet, and cannot be made publically. According to the General Data Protection Regulation, the Swedish law SFS 2018:218, the Swedish Data Protection Act, the Swedish Ethical Review Act, and the Public Access to Information and Secrecy Act, data can only be made available, after legal review, for researchers who meet the criteria for access to this type of confidential data. Readers may contact Professor Kristina Alexanderson (kristina.alexanderson@ki.se).

ORCID iDs

Veronica Svärd https://orcid.org/0000-0002-3868-0254

Kristina Alexanderson https://orcid.org/0000-0002-9313-3413

Figure captions

Figure 1. Proportion (%) of physicians who used the sickness certification guidelines with different frequencies (X-axis), by how often they had sick-leave cases (Y-axis).

Figure 2. Proportions (%) of physicians who stated it was problematic to apply the sickness certification guidelines, by how often they used the guidelines.

For peer review only - http://bmjopen.bmj.com/site/about/guidelines.xhtml

REFERENCES

1 The Swedish Social Insurance Agency. Social Insurance in Figures 2020. Availiable: https://www.forsakringskassan.se/wps/wcm/connect/cf56c741-1668-43c2-887d-10dd6c243cde/socialforsakringen-i-siffror-2020-engelsk.pdf?MOD=AJPERES&CVID= (Accessed 20 March 2021).

2 Söderberg E, Lindholm C, Kärrholm J, et al. Physician's sickness certification practices: A systematic review: Division of Insurance Medicine, Karolinska Institutet, 2019. ISBN 978-91-7831-362-4.

3 Ljungquist T, Hinas E, Nilsson G, et al. Problems with sickness certification tasks: experiences from physicians in different clinical settings. A cross-sectional nationwide study in Sweden. *BMC Health Serv Res* 2015;15:321.

4 Wynne-Jones G, Mallen C, Main C, et al. What do GPs feel about sickness certification? A systematic search and narrative review. *Scand J Prim Health Care* 2010;28:67-75.

5 Winde L, Alexanderson K, Carlsen B, et al. General practitioners' experiences with sickness certification: A comparison of survey data from Sweden and Norway. *BMC Fam Pract* 2012;13:10.

6 Wahlstrom R, Alexanderson K. Swedish Council on Technology Assessment in Health Care (SBU). Chapter 11. Physicians' sick-listing practices (review). *Scand J Public Health Suppl* 2004;63:222-55.

7 Hinkka K, Niemelä M, Autti-Rämö I, et al. Physicians' experiences with sickness absence certification in Finland. *Scand J Public Health* 2018;47(8):859-866.

8 de Boer WE, Mousavi SM, Delclos GL, et al. Expectation of sickness absence duration: a review on statements and methods used in guidelines in Europe and North America. *Eur J Public Health* 2015;26(2):306-11.

9 Letrilliart L, Barrau A. Difficulties with the sickness certification process in general practice and possible solutions: a systematic review. *Eur J Gen Pract* 2012;18(4):219-28.

10 Reed P, editor. *The Medical Disability Advisor. Workplace Guidelines for Disability Duration*. Singapore: Reed Group Holdings, Ltd., 2004.

11 Skånér Y, Nilsson G, Arrelöv B, et al. Use and usefulness of guidelines for sickness certification: results from a national survey of all general practitioners in Sweden. *BMJ Open* 2011;1(2):e000303.

12 The Swedish Board of Health and Welfare. Försäkringsmedicinskt beslutsstöd - vägledning för sjukskrivning (reviderad 2012) (Sickness certification guidelines; revised 2012) (In Swedish), 2012. Availiable: https://roi.socialstyrelsen.se/fmb (Accessed 20 March 2021).

13 World Health Organization. International classification of functioning, disability and health (ICF), 2007. Available: https://www.who.int/standards/classifications/international-classification-offunctioning-disability-and-health (Accessed 20 March 2021)

14 Gustavsson C, Hinas E, Ljungquist T, et al. General practitioners' use of sickness certification guidelines in Sweden at introduction and four years later: a survey study. *Int J Qual Health Care* 2018;30(6):429-38.

15 Gustavsson C, Hinas E, Ljungquist T, et al. Obstetricians/Gynecologists' Problems in Sickness Certification Consultations: Two Nationwide Surveys. *Obstet Gynecol Int* 2016(Article ID 9421316):1-11.

16 Nilsing E, Söderberg E, Öberg B. Sickness certificates in Sweden: did the new guidelines improve their quality? *BMC Public Health* 2012;12(907):1-9.

17 Engblom M, Alexanderson K, Rudebeck CE. Physicians' messages in problematic sickness certification: a narrative analysis of case reports. *BMC Fam Pract* 2011;12:18.

18 Lindholm C, von Knorring M, Arrelöv B, et al. Health care management of sickness certification tasks: results from two survey to physicians. *BMC Res Notes* 2013;6(207):1-7.

For peer review only - http://bmjopen.bmj.com/site/about/guidelines.xhtml

19 Engblom M, Nilsson G, Arrelov B, et al. Frequency and severity of problems that general practitioners experience regarding sickness certification. *Scand J Prim Health Care* 2011;29(4):227-33.
20 Alexanderson K, Arrelöv B, Friberg E, et al. *Läkares erfarenheter av arbete med sjukskrivning av patienter. Resultat från en enkät år 2017 och jämförelser med resultat från motsvarande enkäter år 2012, 2008 respektive 2004. Huvudrapport 2018*. (Physician's experiences of handling sickness certification of patients. Results from 2017 questionnaire and comparisons with results from 2012, 2008, and 2004. Main report.) (In Swedish): Division of Insurance Medicine, Karolinska Institutet, 2018:1-265. ISBN 978-91-7676-054-3. Availiable: https://ki.se/sites/default/files/migrate/2018/03/08/huvudrapport_lakarenkat_2017_2012_2008_2004._ki.pdf (Accessed 21 March 2021).
21 Soderberg E, Alexanderson K. Sickness certification practices of physicians: a review of the literature. *Scand J Public Health* 2003;31(6):460-74.
22 Ljungquist T, Alexanderson K, Kjeldgård L, et al. Occupational health physicians have better work conditions for handling sickness certification compared with general practitioners: Results from a nationwide survey in Sweden. *Scand J Public Health* 2015;43(1):35-43.
23 Roope R, Parker G, Turner S. General practitioners' use of sickness certificates. *Occup Med* 2009;59(8):580-5.
24 Nordhagen H, Harvey S, Rosvold E, et al. Case-specific colleague guidance for general practitioners' management of sickness absence. *Occup Med* 2017;67(8):644-47.

For peer review only

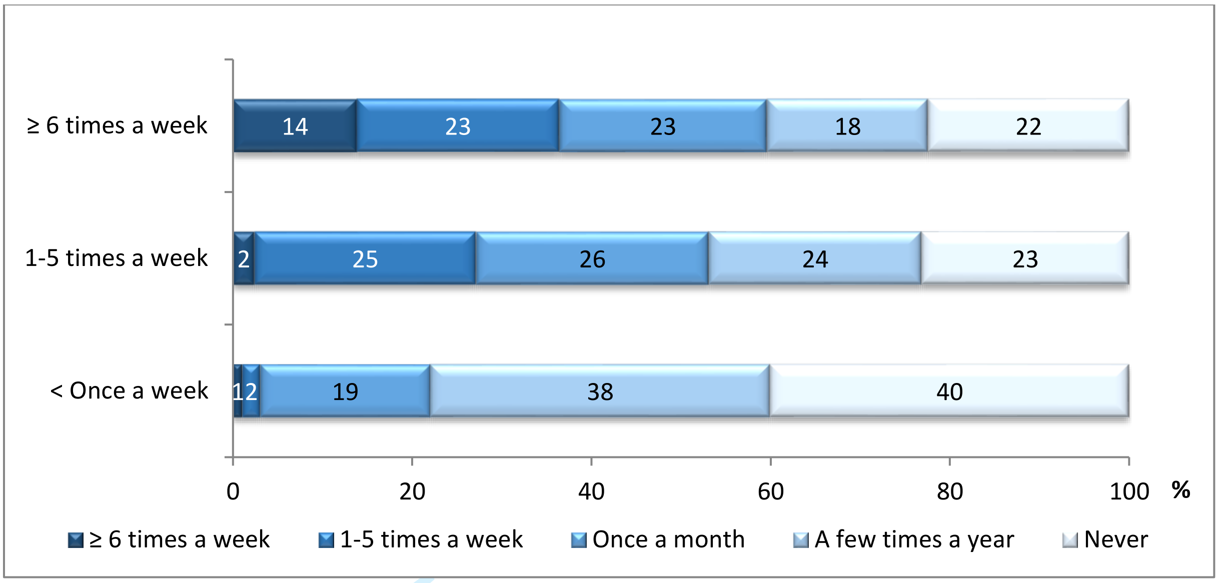

Figure 1. Proportion (%) of physicians who used the sickness certification guidelines with different frequencies (X-axis), by how often they had sick-leave cases (Y-axis).

For peer review only - http://bmjopen.bmj.com/site/about/guidelines.xhtml

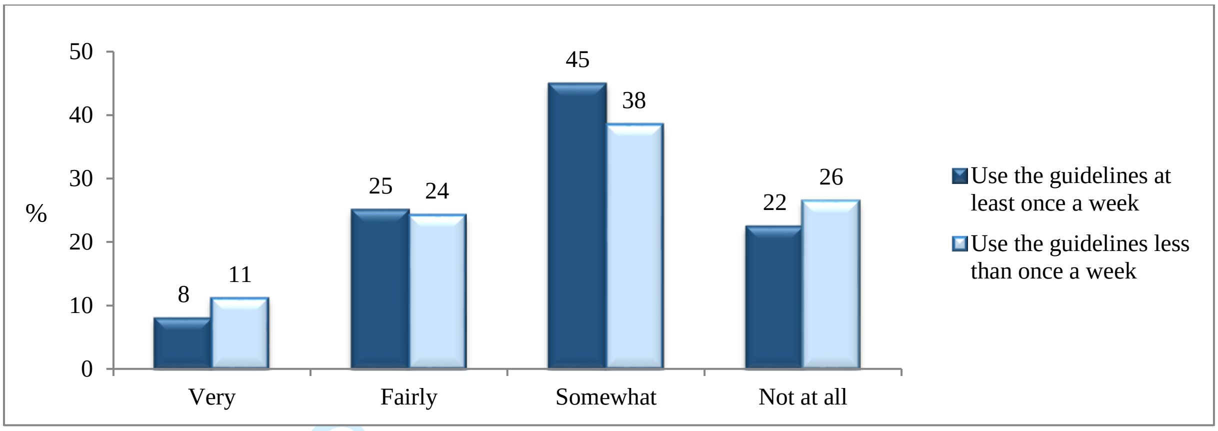

Figure 2. Proportions (%) of physicians who stated it was problematic to apply the sickness certification guidelines, by how often they used the guidelines.

For peer review only

STROBE 2007 (v4) Statement—Checklist of items that should be included in reports of *cross-sectional studies*

Section/Topic	Item #	Recommendation	Reported on page #
**Title and abstract**	1	(*a*) Indicate the study's design with a commonly used term in the title or the abstract	2
		(*b*) Provide in the abstract an informative and balanced summary of what was done and what was found	2
**Introduction**			
Background/rationale	2	Explain the scientific background and rationale for the investigation being reported	4-6
Objectives	3	State specific objectives, including any prespecified hypotheses	6
**Methods**			
Study design	4	Present key elements of study design early in the paper	6
Setting	5	Describe the setting, locations, and relevant dates, including periods of recruitment, exposure, follow-up, and data collection	6-7
Participants	6	(*a*) Give the eligibility criteria, and the sources and methods of selection of participants	7
Variables	7	Clearly define all outcomes, exposures, predictors, potential confounders, and effect modifiers. Give diagnostic criteria, if applicable	8
Data sources/ measurement	8*	For each variable of interest, give sources of data and details of methods of assessment (measurement). Describe comparability of assessment methods if there is more than one group	8
Bias	9	Describe any efforts to address potential sources of bias	*7*
Study size	10	Explain how the study size was arrived at	8
Quantitative variables	11	Explain how quantitative variables were handled in the analyses. If applicable, describe which groupings were chosen and why	8
Statistical methods	12	(*a*) Describe all statistical methods, including those used to control for confounding	8
		(*b*) Describe any methods used to examine subgroups and interactions	8
		(*c*) Explain how missing data were addressed	8
		(*d*) If applicable, describe analytical methods taking account of sampling strategy	N.A.
		(*e*) Describe any sensitivity analyses	N.A.
**Results**			

For peer review only

Participants	13*	(a) Report numbers of individuals at each stage of study—eg numbers potentially eligible, examined for eligibility, confirmed eligible, included in the study, completing follow-up, and analysed	8-9
		(b) Give reasons for non-participation at each stage	7
		(c) Consider use of a flow diagram	N.A.
Descriptive data	14*	(a) Give characteristics of study participants (eg demographic, clinical, social) and information on exposures and potential confounders	8
		(b) Indicate number of participants with missing data for each variable of interest	8
Outcome data	15*	Report numbers of outcome events or summary measures	7-8
Main results	16	(*a*) Give unadjusted estimates and, if applicable, confounder-adjusted estimates and their precision (eg, 95% confidence interval). Make clear which confounders were adjusted for and why they were included	14
		(*b*) Report category boundaries when continuous variables were categorized	N.A.
		(*c*) If relevant, consider translating estimates of relative risk into absolute risk for a meaningful time period	Not relevant
Other analyses	17	Report other analyses done—eg analyses of subgroups and interactions, and sensitivity analyses	N.A.
**Discussion**			
Key results	18	Summarise key results with reference to study objectives	14
Limitations	19	Discuss limitations of the study, taking into account sources of potential bias or imprecision. Discuss both direction and magnitude of any potential bias	16-17
Interpretation	20	Give a cautious overall interpretation of results considering objectives, limitations, multiplicity of analyses, results from similar studies, and other relevant evidence	16-17
Generalisability	21	Discuss the generalisability (external validity) of the study results	17
**Other information**			
Funding	22	Give the source of funding and the role of the funders for the present study and, if applicable, for the original study on which the present article is based	18

*Give information separately for cases and controls in case-control studies and, if applicable, for exposed and unexposed groups in cohort and cross-sectional studies.

Note: An Explanation and Elaboration article discusses each checklist item and gives methodological background and published examples of transparent reporting. The STROBE checklist is best used in conjunction with this article (freely available on the Web sites of PLoS Medicine at http://www.plosmedicine.org/, Annals of Internal Medicine at http://www.annals.org/, and Epidemiology at http://www.epidem.com/). Information on the STROBE Initiative is available at www.strobe-statement.org.

For peer review only
